# Supramolecular encapsulation of indigo with β-cyclodextrin and Hydroxypropyl-β-cyclodextrin: a sustainable strategy for enhanced solubility, stability, and reducing agent-free dyeing processes

**DOI:** 10.1186/s40643-026-01065-w

**Published:** 2026-05-13

**Authors:** Koijam Monica Devi, Nimya Krishnan, Chan-Seo Yeo, Kwon-Young Choi

**Affiliations:** 1 Department of Molecular Science and Technology, Ajou University, Suwon, Gyeonggi-do Republic of Korea; 2 College of Advanced Bio-Convergence Engineering, Ajou University, Suwon, Gyeonggi-do Republic of Korea

**Keywords:** Indigo, β‑cyclodextrin, Hydroxypropyl‑β‑cyclodextrin, Inclusion complex, Solubility enhancement, Reducing agent-free dyeing

## Abstract

Indigo, a ubiquitous vat dye in denim manufacturing, is characterized by its exceptionally low aqueous solubility, necessitating chemical reduction to its leuco form using hazardous agents such as sodium dithionite. This conventional process yields sulfite, sulfate, and sulfide byproducts, leading to significant environmental and toxicological concerns. To address these limitations, this study synthesized 1:1 molar ratio inclusion complexes of indigo with β-cyclodextrin (β-CD) and hydroxypropyl-β-cyclodextrin (HP-β-CD) to enhance solubility and stability without chemical intervention. The formation of these complexes was rigorously validated through UV–Vis, FT-IR, PXRD, TEM, and ^1^H-NMR spectroscopy. Notably, the aqueous solubility of indigo was enhanced 3.58-fold and 5.55-fold for the β-CD and HP-β-CD complexes, respectively. Furthermore, both complexes demonstrated superior thermal and photostability, with HP-β-CD exhibiting the most pronounced effects. This cyclodextrin-assisted solubilization was successfully extended to naturally extracted Jeju Indigo, underscoring its broad applicability. Our findings suggest that cyclodextrin-based encapsulation offers a sustainable and effective alternative to conventional reduction-oxidation dyeing processes.

**Graphical abstract:**

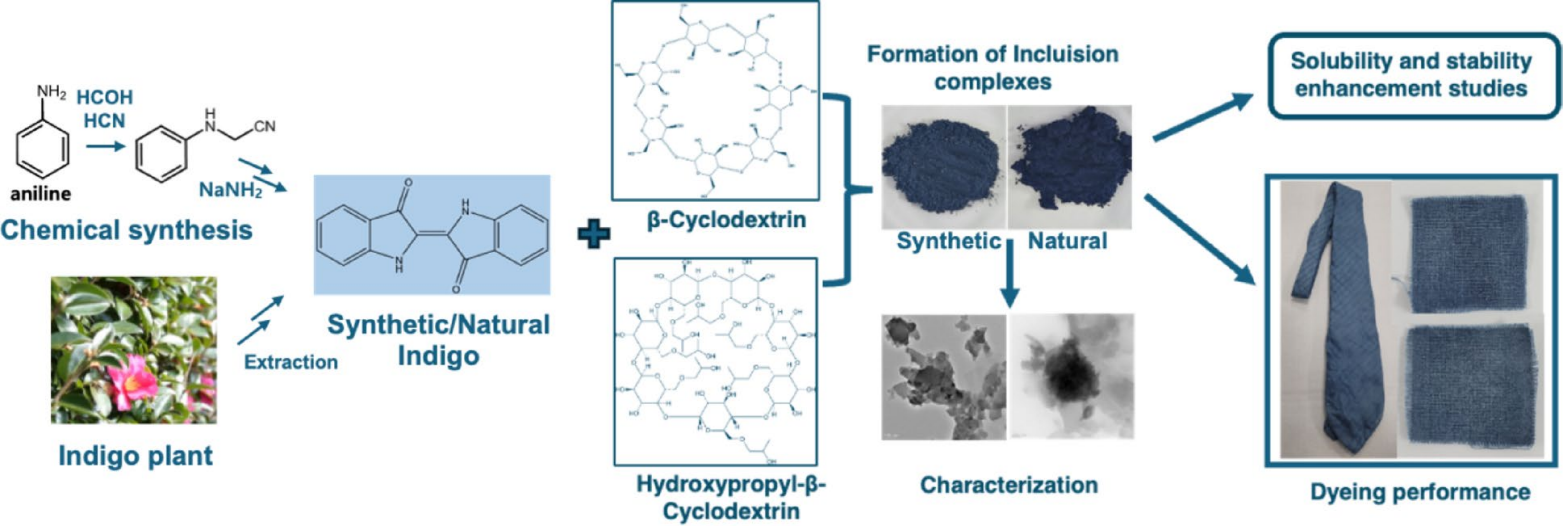

**Supplementary Information:**

The online version contains supplementary material available at 10.1186/s40643-026-01065-w.

## Introduction

Indigo stands as one of the most historically and extensively utilized vat dyes, traditionally derived from plants of the *Indigofera*, *Polygonum*, and *Isatis* genera (Choi 2020). Its distinctive deep blue hue is indispensable for the coloration of cellulosic fibers, particularly within the denim industry. However, its inherent insolubility in water necessitates chemical reduction to a water-soluble leuco form prior to application. Conventionally, this reduction is achieved using sodium dithionite (Na_2_S_2_O_4_), a process that generates byproducts such as sulfite (SO_4_^2−^), sulfate (SO_4_^2−^), and sulfide (S^2−^) (Saikhao et al. 2018). These compounds are known to facilitate the formation of reactive oxygen species (ROS), thereby contributing to environmental degradation and posing significant health risks (Kabish et al. 2023).

In response to these concerns, several alternative reduction strategies have been explored. Electrochemical reduction systems employing iron-based complexes, such as Fe-TEOA-Ca, can reduce chemical waste but demand large electrode surface areas and high energy input (Yi et al. 2020). Organic reducing agents, including α-hydroxyketones, have also been investigated; however, they typically require strongly alkaline conditions and demonstrate limited efficiency (Meksi et al. 2012; Saikhao et al. 2018). Thus, the development of an environmentally sustainable and effective approach for improving indigo solubility and stability remains a critical challenge.

Cyclodextrins (CDs) are cyclic oligosaccharides composed of D-glucopyranose units linked by α-1,4-glycosidic bonds (Sambasevam et al. 2013). Their truncated cone-shaped structure features a hydrophobic internal cavity and a hydrophilic exterior, enabling the encapsulation of hydrophobic guest molecules to form inclusion complexes. Among α-, β-, and γ‑cyclodextrins, β‑cyclodextrin (β‑CD) is most widely used, because its cavity size (~ 0.78 nm) is well-suited for accommodating molecules with molecular weights of 200–800 g/mol (Semcheddine et al. 2015; Butnariu et al. 2021). Hydroxypropyl‑β‑cyclodextrin (HP‑β‑CD), a β‑CD derivative, exhibits enhanced aqueous solubility owing to hydroxypropyl substitution (Halder et al. 2023).

Cyclodextrins (CDs) are widely applied across diverse fields, including pharmaceuticals (e.g., Cannabidiol (Li et al. 2021), Palbociclib (Paul et al. 2024)), food and beverages (β- carotene (Yazdani et al. 2022), laccaic acid (Liu et al. 2017)), and the textile and dye industries (e.g., Vat Red 13 (e.g., Vat Red 13 (Hakeim et al. 2025), azo dyes(Dardeer)). These broad applications are attributed to their low cost and favorable safety profile. Notably, CDs have been shown to enhance dye distribution, supporting environmentally sustainable dyeing practices by reducing the reliance on harmful agents or additives. For instance, Ferriera et al. (2020) investigated the dyeing of polyamide 6 microfiber using a dispersed dye complexed with CDs, reporting that complexation alters dyeing kinetics while improving distribution within the fiber. Similarly, Bezerra et al. (2022) observed improved dyeing of polyamide fibers with Disperse Yellow 211 when complexed with β-CD. Savarino et al. (2004) also noted enhanced color uniformity in nylon fibers dyed in the presence of CDs. Furthermore, Ghoul et al. (2010) demonstrated that polypropylene textiles crosslinked with β-CD exhibit superior dye absorption and retention, while grafting β-CD onto silk threads enhances natural dye adsorption and color stability (Abkenar et al. 2021) .

The capacity of CDs to improve the solubility, stability, and dispersion of hydrophobic compounds underpins their broad application in pharmaceuticals, food, cosmetics, and textiles. In textile dyeing, CDs enhance dye uniformity and fiber affinity while facilitating processes that reduce or replace hazardous additives. These attributes position β-CD and HP-β-CD as promising candidates for forming inclusion complexes with indigo, potentially offering a pathway toward sustainability, reducing agent-free dyeing practices.

**Fig. 1:**
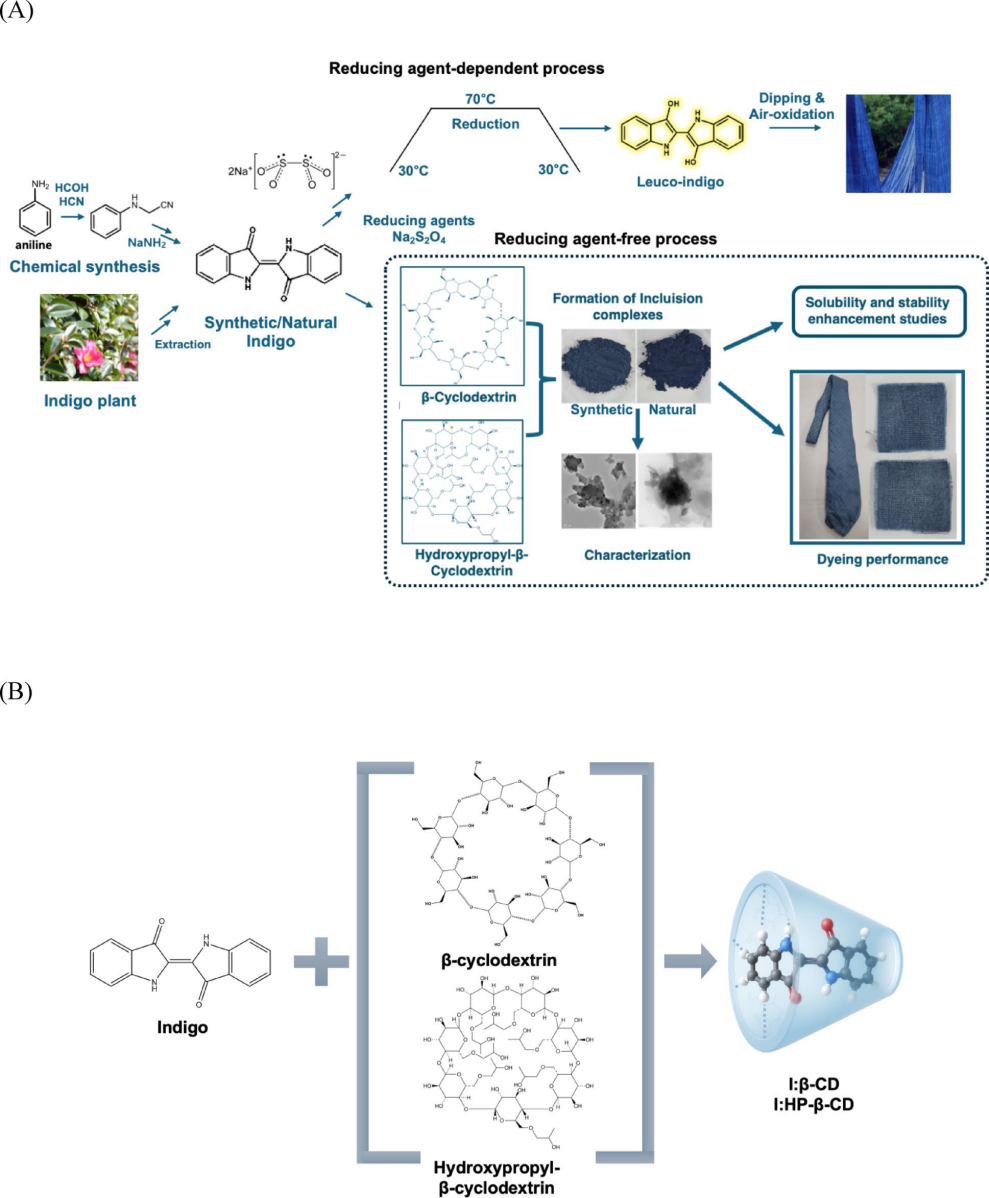
(**A**) Comparison of the general reducing agent-dependent bioprocess and the CD inclusion complex-based,reducing agent-free bioprocess, (**B**) Schematic representation of I:β-CD and I:HP-β-CD inclusion complex synthesis. I:β-CD, indigo:β-cyclodextrin; I:HP-β-CD, indigo:hydroxypropyl-β-cyclodextrin

Consequently, this study aims to enhance the aqueous solubility and stability of indigo dye by forming inclusion complexes with β-CD and HP-β-CD. It also investigates a water-based dyeing approach to reduce reliance on harmful chemical reducing agents, representing a significant advancement in environmentally sustainable textile processing. Here, two types of indigo were used for complex formation with β-CD and HP-β-CD, which includes chemically synthesized indigo and natural indigo extracted from indigo plant grown on Jeju Island,South Korea. Biosynthetic indigo was also tested but did not show distinct difference with chemically synthesized indigo. The general reducing agent-dependent bioprocess and the CD inclusion complex-based, reducing agent-free bioprocess are compared in Fig. 1A. The inclusion complexes were prepared using co-precipitation and kneading methods (Fig. 1B). And the complexes were characterized via UV–Visible spectroscopy, Fourier-transform infrared (FT-IR) spectroscopy, powder X-ray diffraction (PXRD), transmission electron microscopy (TEM), and proton nuclear magnetic resonance (^1^H NMR) to confirm complex formation and evaluate their physicochemical properties.

## Materials and methods

### Materials

Indigo (C_16_H_10_N_2_O_2_; synthetic dye content ≥ 95%, Sigma-Aldrich, CAS No. 482-89-3), β-CD (C_42_H_70_O_35_; purity ≥ 99%, TCI, CAS No. 7585-39-9), and HP-β-CD (C_63_H_112_O_42_; TCI, CAS No. 128446-35-5) were of analytical grade and used without further purification. Dimethyl sulfoxide (DMSO, 99.9%, HPLC grade, Duksan Chemicals) and deionized water were used as solvents throughout the experiments. Natural indigo was kindly provided by Jejuindi Co., Ltd. (Jeju Island, South Korea).

### Phase solubility study

The phase solubility of indigo with β-CD and HP-β-CD was evaluated following the method described by Higuchi and Connors (1965). Excess indigo was added to aqueous solutions of β-CD (0–12 mM) and HP-β-CD (0–30 mM) in conical flasks. The mixtures were shaken at 200 rpm and 30 °C for 48–72 h. Thereafter, the suspensions were centrifuged at 6000 rpm for 8 min, and the supernatants were filtered to remove undissolved indigo. The concentration of dissolved indigo was quantified using UV–Visible spectroscopy. All experiments were performed in triplicate.

Phase solubility diagrams were constructed by plotting the concentration of dissolved indigo against the concentration of CD. The apparent stability constant (K_s_) of each CD complex was determined from the slope of the linear portion of the phase solubility curve using Eq. (1):

1 $$\:{K}_{s}=\frac{Slope}{{S}_{o}\left(1-slope\right)}$$

where S_o_ denotes the intrinsic solubility of indigo in the absence of CD.

### Preparation of inclusion complexes

Inclusion complexes of indigo with β-CD and HP-β-CD were prepared using co-precipitation and kneading methods. In the co-precipitation method (Wang et al. 2011), with slight modifications, equimolar amounts (0.01 mmol, 1:1 ratio) of indigo and CD (β-CD or HP-β-CD) were accurately weighed. CDs were dissolved in 40 mL of deionized water, while indigo was dissolved in 1–2 mL of DMSO. The indigo solution was then added dropwise to the CD solution under continuous stirring. The mixture was sonicated for 30–60 min and then magnetically stirred overnight at 25 °C. The resulting suspensions were then evaporated to dryness under a fume hood. The solid complexes obtained were collected in powder form and stored in airtight glass containers. For the kneading method (Khatun et al. 2023), equimolar amounts of indigo and CD (1:1 molar ratio) were weighed and triturated with a small volume of deionized water using a mortar and pestle to form a homogeneous paste. The paste was then dried in an oven at 40 °C for 30 min. The dried powder was collected and stored in airtight glass containers.

Naturally extracted indigo obtained from Jejuindi Co., Ltd. (Jeju Island, South Korea) was additionally used to prepare HP-β-CD inclusion complexes following the procedures described above. Unless otherwise stated, identical molar ratios and processing conditions were applied.

### Characterization of Indigo: β-cyclodextrin and Indigo: hydroxypropyl-β-cyclodextrin inclusion complexes

The formation of indigo-CD inclusion complexes was analyzed using UV–Visible spectroscopy (Kim 2020). Absorption spectra were obtained with a UV–Visible spectrophotometer (BioTek Epoch Microplate Spectrophotometer, USA) equipped with UV-transparent 96-well microplates, over a wavelength range of 200–800 nm. FT-IR spectra of indigo, β-CD, HP-β-CD, and their inclusion complexes were recorded using a Nicolet iS50 FT-IR spectrophotometer. Spectra were collected over the range of 4000 –400 cm⁻¹ at a resolution of 4 cm^−1^ (Li et al. 2021). PXRD patterns were obtained using a Bruker D8 Advance diffractometer with Cu Kα radiation (λ = 1.5406 Å). Data were recorded over a 2θ range of 5–90° with a step size of 0.02° (Li et al. 2021). TEM images were acquired using a Tecnai G2 F30 S-Twin TEM operating at 300 kV to achieve high-resolution imaging of the samples (Hakeim et al. 2025). ^1^H NMR spectra were recorded at 25 °C on a Bruker Avance III HD spectrometer operating at 600 MHz (Li et al. 2021).

### Water solubility studies

Water solubility was assessed by suspending 20 mg of each sample in 20 mL of deionized water in a beaker and stirring magnetically at 25 °C overnight. The suspensions were then centrifuged at 6000 rpm for 8 min to remove undissolved indigo. The supernatants were filtered, and the concentration of dissolved indigo was measured at 620 nm using a UV–Visible spectrophotometer. Solubility measurements for inclusion complexes prepared with naturally extracted Jeju indigo were conducted under identical conditions.

### Stability analysis

The stability of indigo and its cyclodextrin (CD) inclusion complexes was evaluated under varying environmental conditions. Suspensions were divided into three groups and stored as follows: (i) at room temperature, with and without light exposure for 1 month (ambient storage); (ii) under continuous UV irradiation at 254 nm using a TN-4 C UV lamp for 1 week (photostability); and (iii) at 100 °C for 1 week (thermal stability). Absorbance was measured at designated time intervals using a UV–Visible spectrophotometer to monitor degradation or stability changes. All experiments were conducted in triplicate (*n* = 3), and data are presented as mean ± standard deviation. Statistical analysis was performed using one-way analysis of variance (ANOVA) followed by Tukey’s HSD test, with significance considered at *p* < 0.05.

### Dyeing and color fastness analysis

Indigo-cyclodextrin (Indigo-CD) suspensions were prepared at a concentration of 5% (w/v) from kneaded inclusion complexes using deionized water. Samples included indigo without cyclodextrin (control), Jeju indigo:hydroxypropyl-β-cyclodextrin (JI: HP-β-CD), Indigo:β-cyclodextrin (I:β-CD), and Indigo:hydroxypropyl-β-cyclodextrin (I:HP-β-CD), all prepared under identical conditions. Cotton fabric samples were pretreated by washing with deionized water and air-dried at room temperature. The fabrics were immersed in the dye suspensions and incubated at 50 °C overnight to facilitate dye uptake. Color evaluation was performed using a JZ-600 colorimeter (Shenzhen Kingwell Instrument Co., Ltd., Guangdong, China) (Ahn et al. 2019).

Color fastness to washing and light exposure was evaluated using CIELAB color space parameters. For washing fastness, dyed fabrics were washed in a detergent solution at 50 °C for 20 min, rinsed with deionized water, and air-dried. For light fastness, samples were exposed to natural sunlight for 7 days under ambient conditions.

Color parameters (L*, a*, b*) were measured before and after treatment using a JZ-600 colorimeter (Shenzhen Kingwell Instrument Co., Ltd., Guangdong, China), and the total color difference (ΔE*ab) was calculated. All measurements were performed in triplicate.

### Dye fixation measurement

Dye fixation (%) was determined based on the color strength (K/S) values of dyed fabrics before and after washing. The K/S values were measured using a CM700d spectrophotometer (Konica Minolta, Japan). Measurements were carried out under the following conditions: K/S mode, measurement wavelength of 620 nm, and a D65 illuminant with a 10° standard observer (Lin et al. 2021; Safi and Amirshahi 2023).

The percentage of dye fixation was calculated using the following Eq. (2):

2 $$ {\mathrm{Fixation}}\,{\mathrm{rate}}\,\left( \% \right)\, = \,\left( {{\mathrm{K}}/{\mathrm{S}}} \right)_{{\mathrm{a}}} /\left( {{\mathrm{K}}/{\mathrm{S}}} \right)_{{\mathrm{b}}} \times {\mathrm{1}}00 $$

where (K/S) _b_ and (K/S) _a_ represent the color strength values of the cotton dyed fabric before and after the washing (soaping) process, respectively. All measurements were performed in triplicate.

## Results and discussions

### Phase solubility studies

**Fig. 2:**
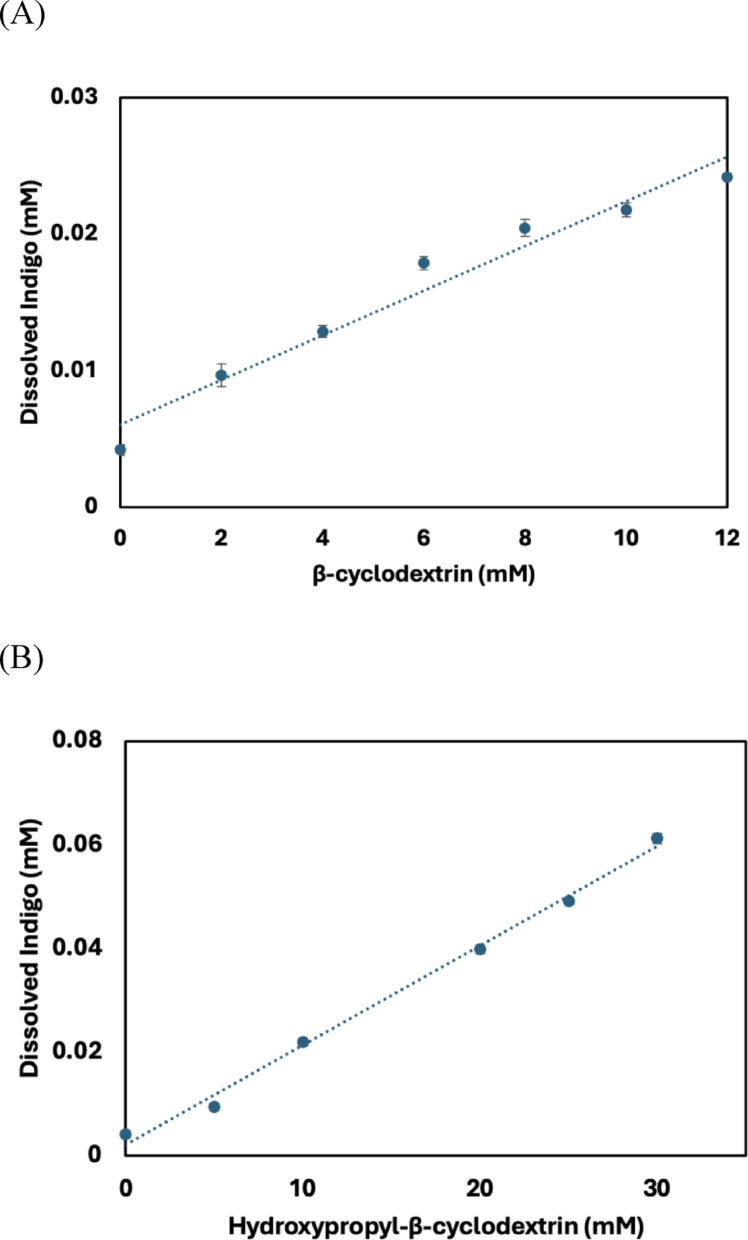
Phase solubility profiles of (**A**) indigo with β-cyclodextrin and (**B**) indigo with hydroxypropyl-β-cyclodextrin at 25 °C

Figure 2 presents the phase solubility profiles of indigo in the presence of β-CD and HP-β-CD. Phase solubility analysis is a standard method for evaluating the solubilizing capacity of CDs toward guest molecules and determining the stoichiometric ratio of the resulting inclusion complexes (Gürten et al. 2018). For both β-CD and HP-β-CD, indigo solubility increased linearly as a function of CD concentration. This observation is consistent with an AL-type diagram according to the Higuchi-Connors classification (Raval and Bagada 2019), thereby indicating a 1:1 stoichiometry for both complexes. The apparent stability constants (Ks), calculated using Eq. (1), were 335 M^−1^ for β-CD and 450 M^−1^for HP-β-CD. Stability constants within the 100–1000 M^−1^range are generally considered optimal for the formation of stable inclusion complexes (Miranda et al. 2011; Santos et al. 2017).

### UV–Visible spectroscopy

**Fig. 3:**
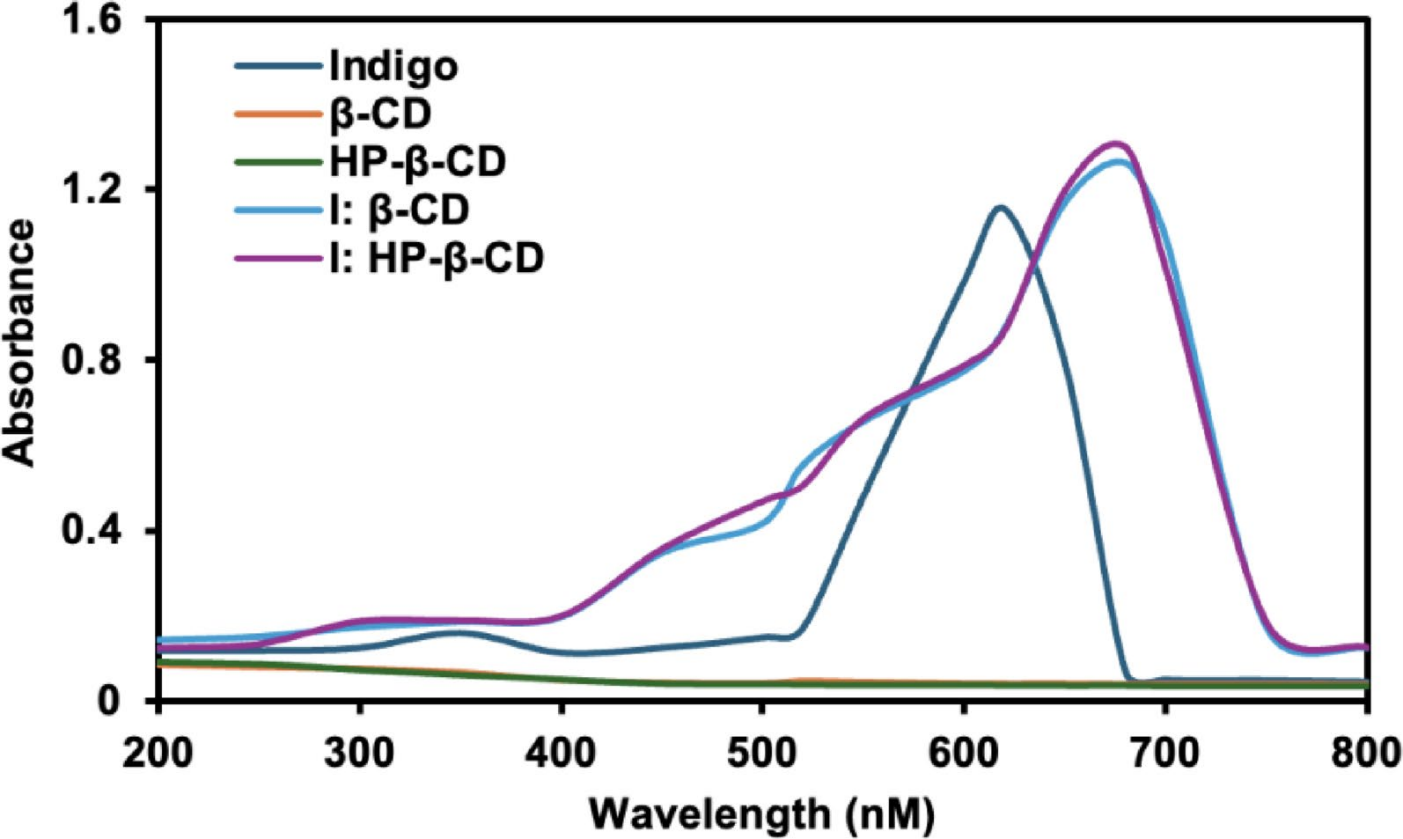
UV–Visible absorption spectra of indigo, β-CD, HP-β-CD, I:β-CD, and I:HP-β-CD via co-precipitation

Figure 3 illustrates the UV–Visible spectra of indigo, β-CD, HP-β-CD, and their respective inclusion complexes. Pure indigo exhibited a maximum absorption peak at approximately 620 nm. In contrast, both β-CD and HP-β-CD showed no significant absorbance in this region, which can be attributed to the absence of π-electrons or non-bonding electrons (Kim 2020). Upon complexation, a distinct bathochromic shift of the maximum absorption wavelength to ~ 670 nm was observed for both inclusion complexes. This redshift serves as evidence of inclusion complex formation and correlates with the enhanced aqueous solubility of indigo. These spectral modifications suggest the effective encapsulation of indigo molecules within the CD cavities, consistent with previously reported host–guest interaction studies (Sarabia-Vallejo et al. 2023).

### Fourier transform infrared analysis

**Fig. 4:**
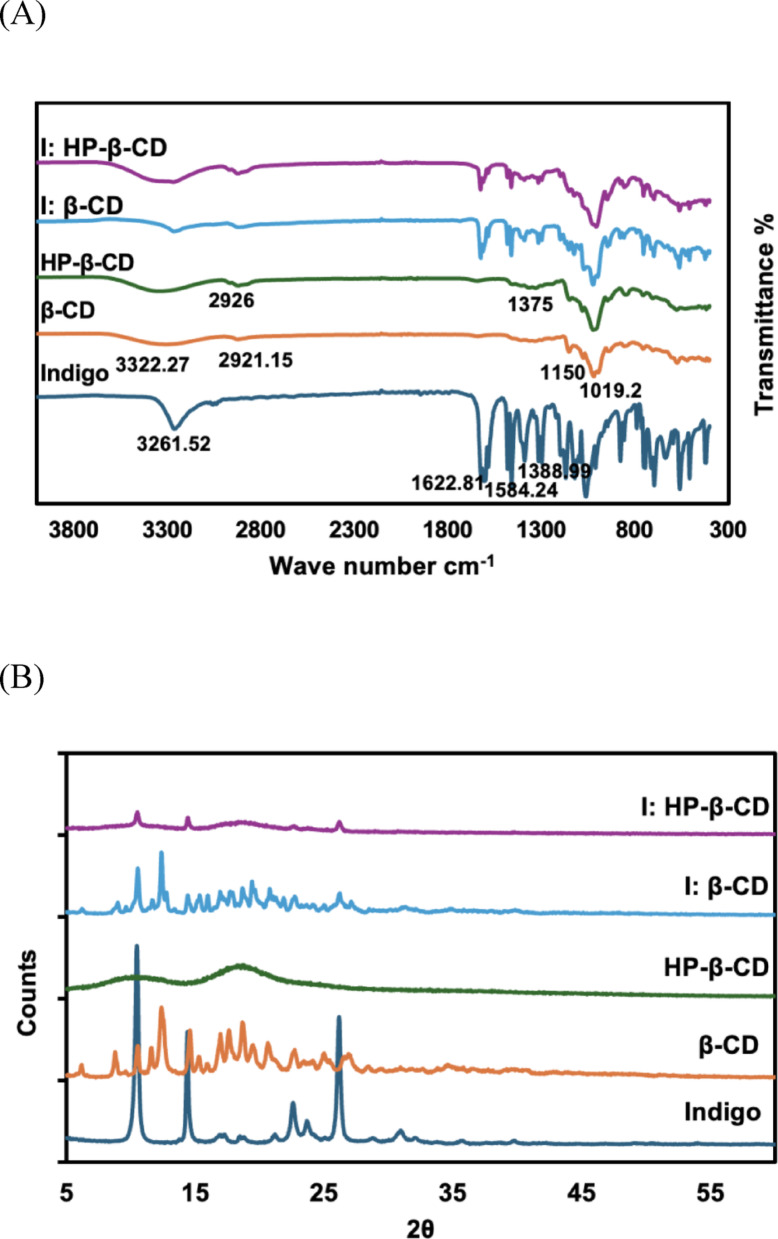
(**A**) FT-IR spectra of indigo, β-CD, HP-β-CD, I:β-CD, and I:HP-β-CD via co-precipitation; (**B**) PXRD patterns of indigo, β-CD, HP-β-CD, I:β-CD, and I:HP-β-CD via co-precipitation

FT-IR spectroscopy was employed to further examine molecular interactions and verify the formation of inclusion complexes (Mura 2015). As shown in Fig. 4A, the FT-IR spectra of pure indigo, β-CD, HP-β-CD, and the resulting I:β-CD and I:HP-β-CD complexes were compared. Pure indigo displayed characteristic absorption bands at 3261.52 cm^−1^ (N–H stretching), 1600.63–1622.81 cm^−1^ (C=O stretching), 1584.24 cm^−1^ (aromatic C=C stretching), and 1388.98 cm^−1^ (C–N stretching), along with a weak aromatic C–H stretching peak at 3057 cm^−1^ (Baran et al. 2010; Ju et al. 2019). The β-CD spectrum exhibited typical peaks at ~ 3322.27 cm^−1^ (O–H stretching), 2921.15 cm^−1^ (C–H stretching), 1150 cm^−1^ (C–O–C stretching), and 1019.2 cm^−1^ (C–O stretching) (Sambasevam et al. 2013). HP-β-CD showed a similar profile, with additional signals near 2965.02 cm^−1^ and 1375 cm^−1^ corresponding to the methyl group vibrations of the hydroxypropyl substituents (Kim 2020). In both inclusion complexes, the characteristic bands of indigo—specifically those related to C–N, C = O, and N–H exhibited reduced intensity, broadening, and significant shifts. These changes reflect the altered chemical environments resulting from molecular encapsulation. Furthermore, modifications in the O–H stretching and C–O–C vibrations of the CDs confirm the establishment of stable host-guest complexes through intermolecular interactions.

### Powder X-ray diffraction analysis

The PXRD patterns of pristine indigo, β-CD, HP-β-CD, and their complexes are presented in Fig. 4B. Pristine indigo exhibited three distinctive diffraction peaks at 2θ values of 10.48°, 14.39°, and 26.13°, confirming its crystalline nature (Kettner et al. 2011; Bouzidi et al. 2017). β-CD displays multiple prominent diffraction peaks at 8.81°, 10.56°, 12.40°, 14.60°, 17.00°, 17.65°, 18.73°, 20.71°, and 22.79°, alongside several smaller peaks reflecting its crystalline framework (Rachmawati et al. 2013). In contrast, HP-β-CD shows only broad peaks, characteristic of an amorphous structure (Kim 2020; Khan et al. 2023). The diffraction pattern of the I:β-CD complex shows significant alterations relative to pristine indigo. The distinct sharp characteristic indigo peaks at the defined 2θ positions decrease in intensity and broaden, indicating a substantial reduction in crystallinity. This suggests that indigo molecules are encapsulated within the hydrophobic cavity of β-CD, disrupting its native crystal lattice, resulting in an amorphous or less-ordered arrangement. Although the characteristic diffraction peaks of indigo show a significant reduction in the intensity of I:β-CD inclusion complex, the diffraction features resemble those of β-CD (Tom et al. 2020), suggesting that encapsulation diminishes indigo crystallinity while the host preserves its crystalline framework, affirming stable complex formation.

In I:HP-β-CD complex, the sharp diffraction peaks of indigo completely disappeared, indicating a significant loss in crystalline order upon complex formation. The hydroxypropyl substituents in HP-β-CD influence amorphous characteristics, yielding a broad diffuse diffraction pattern compared to that of the I:β-CD complex. These findings confirm that indigo molecules are encapsulated within the cyclodextrin cavity, disrupting the molecular arrangement of the dye.

### Transmission electron microscope analysis

**Fig. 5:**
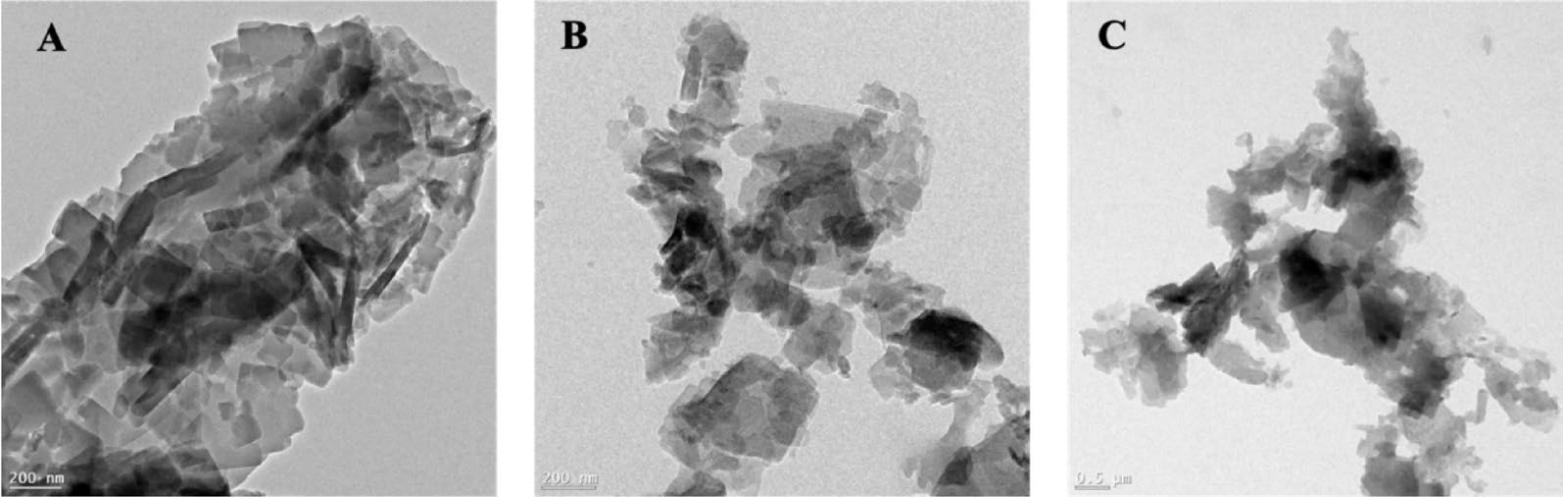
TEM images of (**A**) indigo; (**B**) I:β-CD, and (**C**) I:HP-β-CD prepared via co-precipitation

TEM was used to visualize the morphological and structural characteristics of the indigo inclusion complex with β-CD, HP-β-CD (Hakeim et al. 2025). Figure 5 shows that pure indigo (A) appears as dense, irregular rod-shaped aggregates, consistent with strong intermolecular π–π interactions and a highly crystalline structure. In contrast, the I:β-CD and I:HP-β-CD complexes (B, C) exhibit smaller, more uniform nanoparticulate morphologies with reduced aggregation. These morphological transitions suggest disruption of the native crystalline structure of indigo, yielding more porous structures as previously reported (Hakeim et al. 2025). These TEM observations are consistent with the PXRD results, which show a marked reduction or complete disappearance of the characteristic crystalline diffraction peaks of indigo upon complex formation. The combined evidence from TEM and PXRD confirms the loss of long-range crystalline order and supports successful inclusion of indigo within the cyclodextrin cavities. Such structural modifications probably enhance aqueous solubility and further confirm the successful encapsulation of indigo within CD cavities.

### Proton nuclear magnetic resonance analysis

Figure S1 shows the ¹H NMR spectrum of pure indigo, with characteristic aromatic proton resonances between 6.9 and 7.64 ppm, primarily corresponding to aromatic C–H protons adjacent to the carbonyl (C = O) groups within the conjugated indole system. Additionally, a broad resonance near 10.5 ppm is attributed to the hydrogen-bonded N–H protons of the cyclic amide (indole) rings (Kuntze et al. 2023). Figure S2 shows that the ^1^H NMR spectrum of β-CD exhibits characteristic signals from its seven glucopyranose units. The anomeric protons (H-1) appeared as distinct peaks at 4.9–5.0 ppm, indicating their involvement in glycosidic linkages. The ring protons H-2,4 (3.44–3.56 ppm), H-3,5,6 (3.72–3.88 ppm) appear as overlapping signals in the spectra, where H-3 and H-5 serve as cavity-facing protons and clear indicators of guest inclusion (Sompornpisut et al.).

Similarly, in the ^1^H NMR spectrum of HP-β-CD (Dufour et al. 2015), the anomeric protons (H-1) of the glucopyranose units typically resonate at 4.9–5.2 ppm. The sugar ring protons (H-2 to H-6) appear as overlapping signals between 3.3 and 4.0 ppm. A characteristic signal for HP-β-CD is the methyl group (−CH_2_) of the hydroxypropyl side chains, appearing as a broad doublet near 1.1 ppm (Figure S3). The I:β-CD inclusion complex prepared via co-precipitation shows new peaks/doublets at 5.65–5.75 ppm, shifted downfield from the free β-CD H-1 peaks (4.9–5.0 ppm), indicating changes in the anomeric proton environment due to indigo complexation (Figure S4). H-3 and H-5 protons show visible changes, such as upfield shifts to lower ppm. In the I:HP-β-CD complex (Figure S5), the H-1 proton shifted downfield to 5.5–6.0 ppm, likely due to an altered environment from indigo inclusion, with slight peak broadening near H-3 and H-5. This suggests host-guest binding alters local electronic environments. However, both complexes still show weaker free indigo peaks, reflecting partial encapsulation or indigo remaining outside the cavity(Kuntze et al. 2023; Sompornpisut et al.). Nevertheless, some indigo molecules are encapsulated within the cyclodextrin cavity, forming host–guest complexes that alter the β-CD and HP-β-CD proton environments.

### Water solubility

**Fig. 6:**
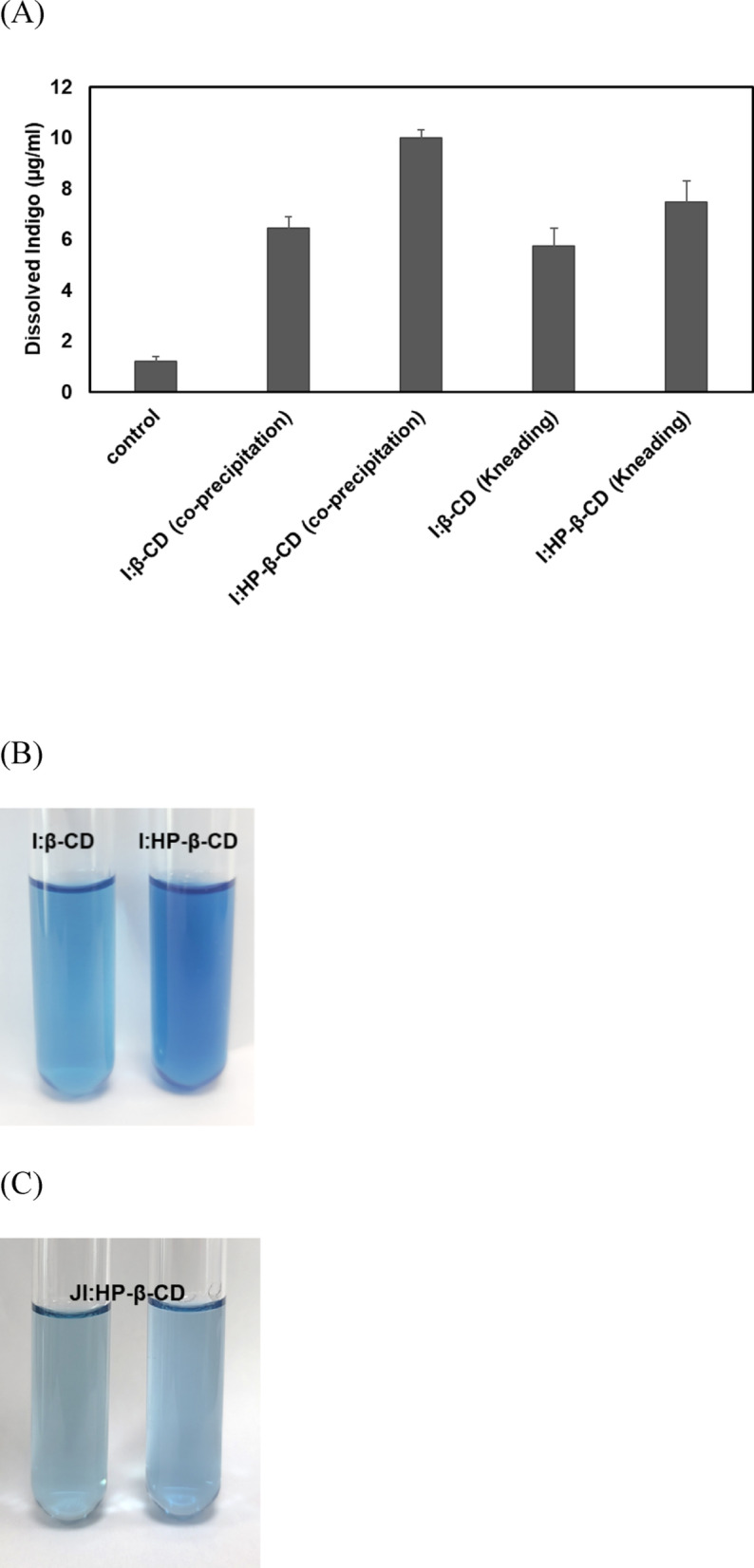
(**A**) Aqueous solubility of indigo (control), I:β-CD, and I:HP-β-CD via co-precipitation, as well as I:β-CD, and I:HP-β-CD via kneading (*n* = 3); (**B**) Visual images of clear dissolved supernatant of I:β-CD, and I:HP-β-CD inclusion complexes via co-precipitation; (**C**) Visual images of clear dissolved supernatant of Jeju Indigo:HP-β-CD inclusion complexes. The inclusion complexes were suspended in deionized water, stirred at 25 °C, centrifuged, and the clear supernatants were used to analyze dissolved indigo concentration

The aqueous solubility of indigo was substantially enhanced when complexed with β-CD and HP-β-CD (Fig. 6A). Control indigo exhibited low solubility in water (1.8 µg/mL). However, co-precipitated inclusion complexes with β-CD and HP-β-CD increased solubility to 6.45 µg/mL and 9.99 µg/mL, respectively, representing 3.58-fold and 5.55-fold enhancements over raw indigo. The kneading method also enhanced solubility to 5.75 µg/mL (I:β-CD) and 7.46 µg/mL (I:HP-β-CD), corresponding to ~ 3.19-fold and 4.14-fold increases, respectively. Overall, co-precipitation yielded greater solubility enhancement than kneading, emphasizing a higher efficiency in forming inclusion complexes (Kani and N Xavier 2021). The photograph in Fig. 6B visually confirms the clarity of dissolved indigo, observed in the clear supernatant obtained after mixing and centrifuging I:β-CD and I:HP-β-CD complexes, prepared via co-precipitation, indicating complete dissolution. In addition, the photograph in Fig. 6C shows natural Jeju indigo:HP-β-CDcomplexes, with the left image corresponding to the kneaded complex and the right image corresponding to the co-precipitated complex, both displaying a clear supernatant after centrifugation, indicating dissolved indigo. Overall, these findings indicate that β-CD and HP-β-CD significantly improve the aqueous solubility of indigo, with HP-β-CD exhibiting the greatest effect.

### Stability performance

All stability tests were performed on the clear dissolved supernatants obtained after co-precipitation complexation to accurately reflect the realities of indigo’s use in textile chemistry, where the substance primarily acts as a dispersed solute rather than a fixed solid. This strategy captures the true challenges faced by indigo in aqueous media during extended storage under ambient lighting, intensive UV irradiation mimicking accelerated weathering, and high-temperature incubation simulating dyeing vat conditions. It underscores the applied benefits of cyclodextrin host-guest interactions in maintaining indigo stability during use. To comprehensively assess the stability of indigo in solution, three complementary tests were conducted on the clear dissolved supernatants of I:β-CD and I:HP-β-CD inclusion complexes prepared via co-precipitation. Dissolved indigo concentration was monitored over time to assess the effects of ambient light, ultraviolet irradiation, and elevated temperature.

**Fig. 7:**
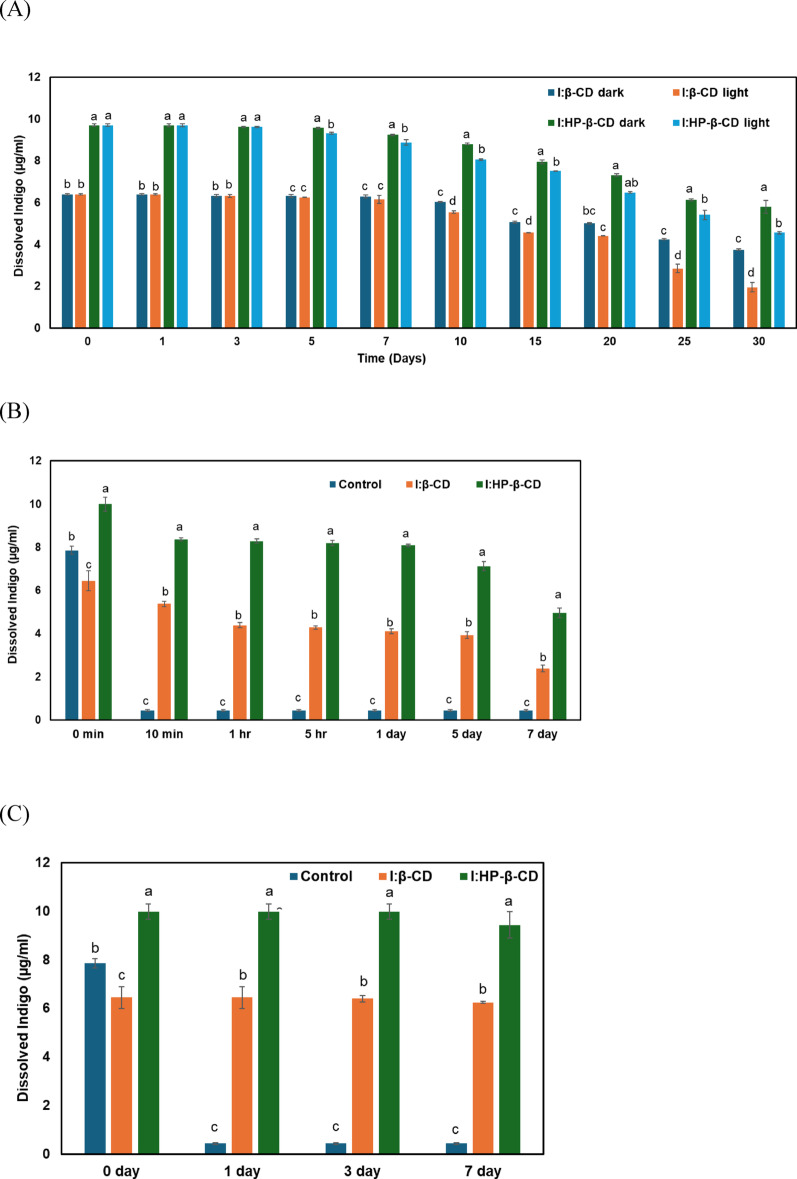
Stability of I:β-CD and I:HP-β-CD inclusion complexes (co-precipitation method) under: (**A**) storage under light and dark conditions at room temperature for 30 days; (**B**) UV irradiation for 7 days, compared with indigo in DMSO (control); (**C**) thermal treatment at 100 °C for 7 days, compared with indigo in DMSO (control). Experiments were performed using clear dissolved supernatants of I:β-CD and I:HP-β-CD inclusion complexes after centrifugation. Data are presented as mean ± standard deviation (*n* = 3). Different letters indicate statistically significant differences among treatments at each time point (one-way ANOVA followed by Tukey’s HSD test, *p* < 0.05)

The complexes were stored under light and dark conditions for 30 days. Both complexes were more stable in the dark than under light exposure (Fig. 7A). At day 30, the I:HP-β-CD complex retained ~ 6.0 µg/mL of dissolved indigo in the dark, whereas the light-exposed sample decreased to ~ 4.5 µg/mL. In contrast, the I:β-CD complex degraded faster under both conditions, with the light-exposed sample falling below 2.5 µg/mL of indigo by day 30. Both complexes were significantly more stable under dark conditions compared to light exposure at later time points (*p* < 0.05).

In the UV stability test (Fig. 7B), DMSO-dissolved indigo served as a control, showing an immediate decrease in indigo concentration within 10 min, indicating photodegradation under UV exposure. However, the inclusion complexes showed minimal photodegradation during the initial stages, as evidenced by the slight decrease in dissolved indigo concentration within the first few hours. During UV exposure, I:β-CD and I:HP-β-CD showed a gradual decline in indigo levels. However, the I:HP-β-CD complex showed a smaller reduction, retaining higher levels of dissolved indigo after 7 days and consistently maintaining significantly higher concentrations than I:β-CD throughout the experiment (*p* < 0.05), indicating superior photostability.

Additionally, the thermal stability of the dissolved complexes was assessed through incubation at 100 °C for 7 days. The control (indigo in DMSO) degraded rapidly, with dissolved indigo concentration decreasing from 7.8 µg/mL on day 0 to below 0.5 µg/mL by day 1 (Fig. 7C). The I:β-CD complex showed moderate stability, maintaining ~ 6.4 µg/mL of dissolved indigo throughout the test period. The I:HP-β-CD complex exhibited excellent thermal stability, retaining nearly 9 µg/mL of indigo even after prolonged incubation, demonstrating significantly improved thermal stability (*p* < 0.05).

This enhanced stability of HP-β-CD likely results from its hydroxypropyl modifications, which enhance amorphization and water compatibility, supporting better preservation of dissolved indigo despite slightly reduced encapsulation strength as observed with NMR. Therefore, although β-CD forms a more defined host-guest complex, HP-β-CD enhances encapsulation, solubility, and stability, making it a more effective host for enhancing indigo stability and solubility.

### Dyeing performance, color fastness and fixation analysis

**Fig. 8:**
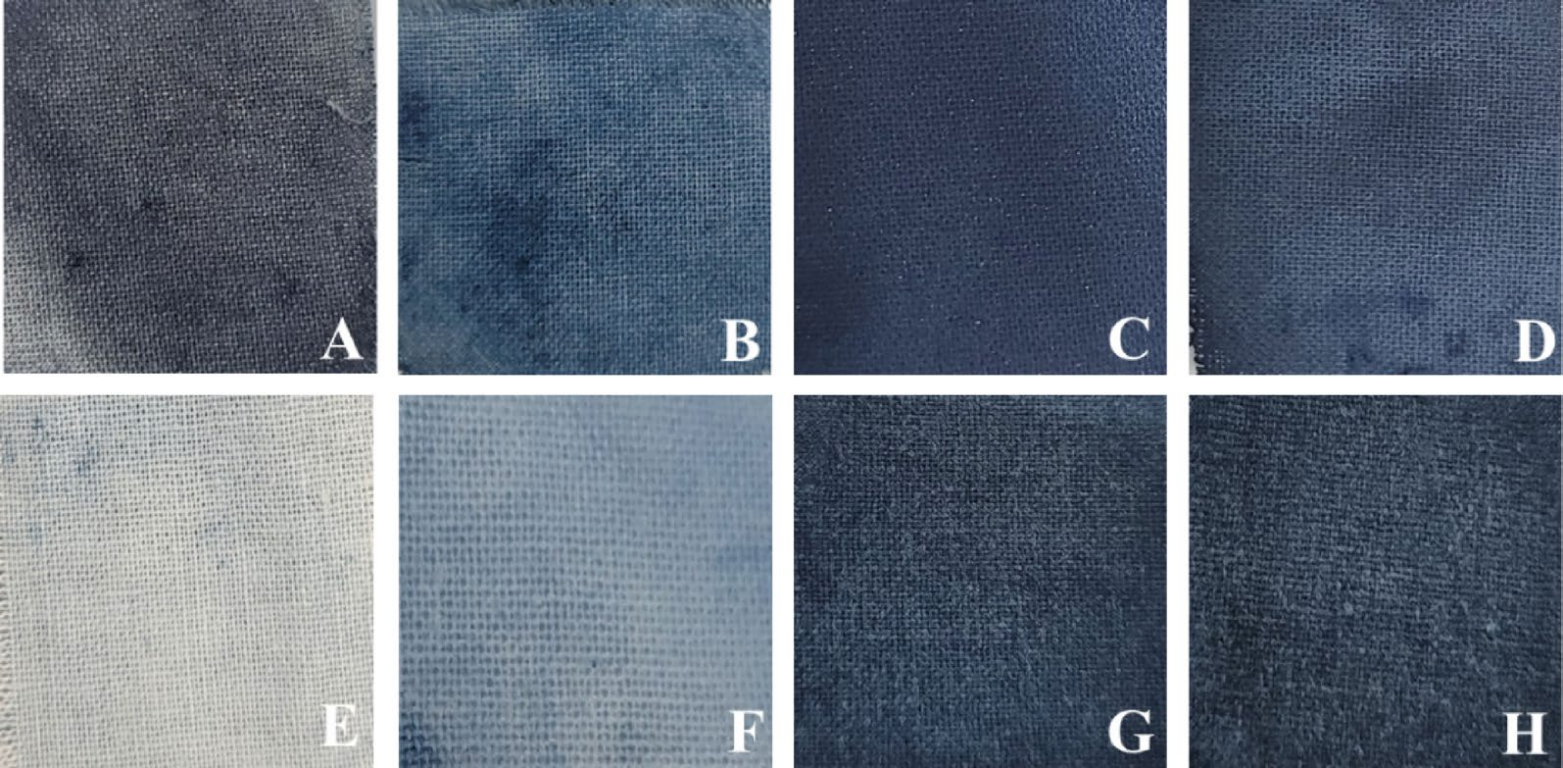
Photographic images of cotton fabrics dyed with indigo and cyclodextrin inclusion complexes (5% w/v): (**A**–**D**) before washing and (**E**–**H**) after washing. Samples are arranged from left to right as indigo dyed without cyclodextrin (control), JI:HP-β-CD, I:β-CD, and I:HP-β-CD, where all cyclodextrin inclusion complexes were prepared via the kneading method, illustrating differences in color retention

The dyeing effectiveness of indigo and indigo-cyclodextrin (CD) inclusion complexes was evaluated on cotton fabrics using 5% (w/v) suspensions prepared via the kneading method. Visual inspection of dyed fabrics (Fig. 8) revealed noticeable differences in color depth and retention among the samples before and after washing. Fabrics treated with cyclodextrin inclusion complexes exhibited darker and more uniform coloration compared to the control.

Colorimetric analysis based on CIELAB parameters (Table 1) showed differences in color properties among the dyed fabrics. Before washing, fabrics treated with I:β-CD and I:HP-β-CD exhibited lower L* values than the control, indicating darker shades. After washing, L* values increased for all samples, reflecting color loss, although the change was less pronounced in inclusion complex-treated fabrics. The ΔE*ab values indicated lower overall color change in samples treated with cyclodextrin inclusion complexes compared to the control, suggesting improved color stability. All samples showed negative b* values, confirming retention of the characteristic blue hue of indigo, with smaller shifts observed in the inclusion complexes after washing.

**Table 1:** Color fastness to washing of cotton fabrics dyed with indigo and cyclodextrin inclusion complexes (5% w/v), evaluated using CIELAB color coordinates (L*, a*, b*) before (B) and after (A) washing and color difference (ΔE*ab)

Samples	L* (B)	a* (B)	b* (B)	L* (A)	a* (A)	b* (A)	ΔE*ab
Control	53.10	− 2.29	− 8.14	69.36	− 5.48	− 7.68	16.58
JI:HPβCD	49.86	− 5.54	− 11.05	61.45	− 5.21	− 8.97	11.78
I:β-CD	20.45	− 2.36	− 10.11	39.78	− 2.11	− 11.80	19.40
I:HPβCD	24.51	− 1.09	− 10.81	44.44	− 2.19	− 11.25	19.97

L*, lightness; a*, red (+) to green (−); b*, yellow (+) to blue (−); ΔE*ab, total color difference; B, before washing; A, after washing; I, indigo; JI, Jeju indigo; β-CD, β-cyclodextrin; HP-β-CD, hydroxypropyl-β-cyclodextrin; Control, indigo dyed without cyclodextrin

ΔE*_ab_ $$\:=\sqrt{{\left({L}_{after}^{*}-{L}_{before}^{*}\right)}^{2}+{\left({a}_{after}^{*}-{a}_{before}^{*}\right)}^{2}+{\left({b}_{after}^{*}-{b}_{before}^{*}\right)}^{2}}$$

**Fig. 9:**
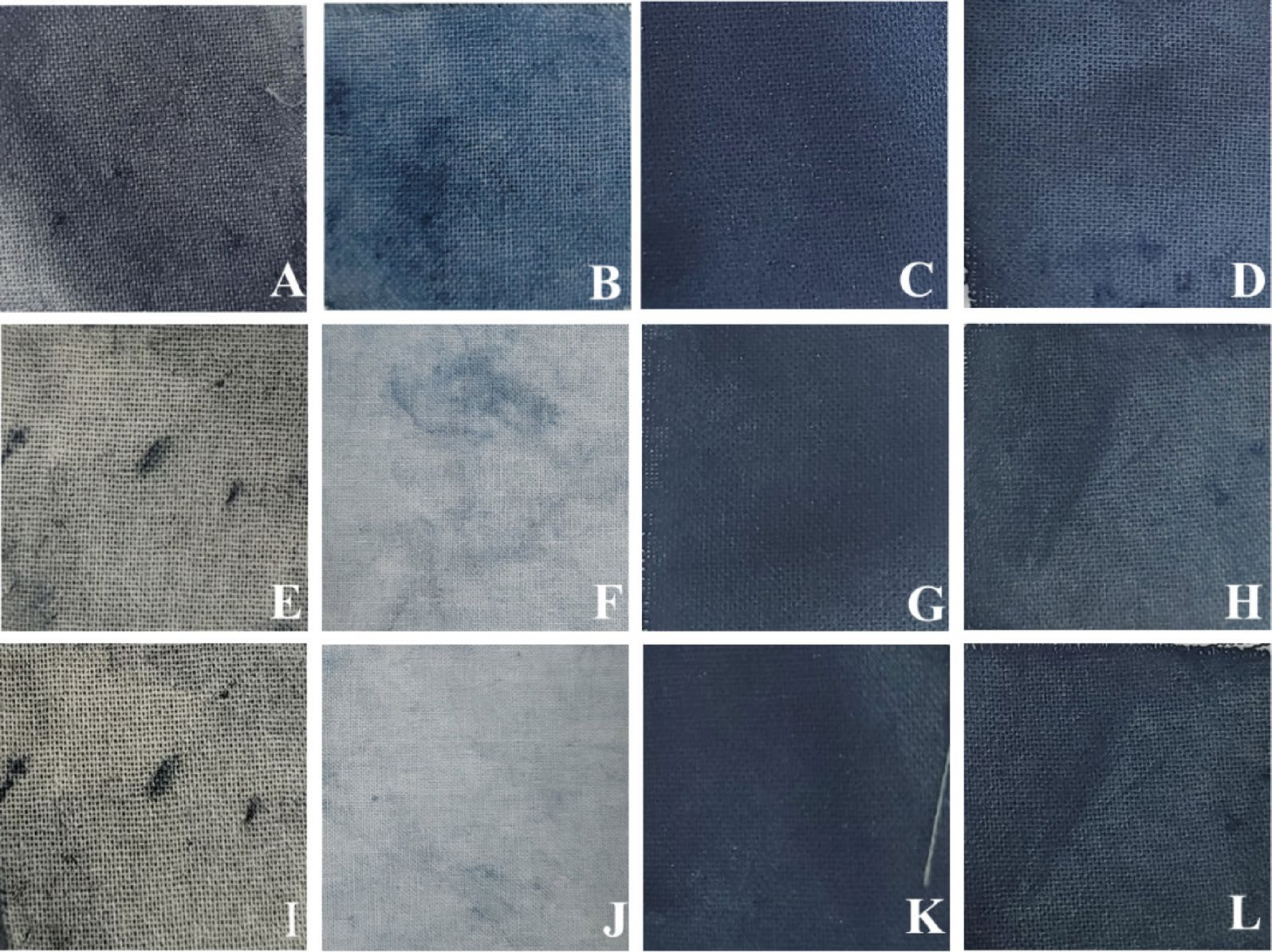
Photographic images of cotton fabrics dyed with indigo and cyclodextrin inclusion complexes (5% w/v) prepared via the kneading method, following sunlight exposure. Samples are arranged row-wise according to exposure time: (**A**–**D**) 0 days, (**E**–**H**) 3 days, and (**I**–**L**) 7 days. Within each row, samples are arranged from left to right as control (indigo dyed without cyclodextrin), JI:HP-β-CD, I:β-CD, and I:HP-β-CD. The images illustrate differences in color retention and photostability of the dyed fabrics over time

Color fastness under natural sunlight exposure further demonstrated the stabilizing effect of cyclodextrin inclusion (Table 2; Fig. 9). After 7 days of exposure, fabrics dyed with CD complexes exhibited significantly lower ΔE*ab values compared to the control, indicating reduced color degradation. Notably, I:HP-β-CD (ΔE*ab = 3.73) and I:β-CD (ΔE*ab = 3.91) showed superior photostability compared to the control (ΔE*ab = 11.41), consistent with visual observations (Fig. 9).

**Table 2:** Color fastness to natural sunlight exposure (7 days) of cotton fabrics dyed with indigo and cyclodextrin inclusion complexes (5% w/v), evaluated using CIELAB color coordinates (L*, a*, b*) measured before (B) and after (A) exposure, and expressed as ΔE*ab

Samples	L* (B)	a* (B)	b* (B)	L* (A)	a* (A)	b* (A)	ΔE*ab
Control	53.1	− 2.29	− 8.14	60.4	− 2.45	0.63	11.41
JI:HPβCD	49.86	− 5.54	− 11.05	57.52	− 5.81	− 6.06	9.15
I:β-CD	20.45	2.36	− 10.11	22.81	− 0.16	− 8.27	3.91
I:HPβCD	24.51	1.09	− 10.81	25.75	− 1.06	− 8.03	3.73

L*, lightness; a*, red (+) to green (−); b*, yellow (+) to blue (−); ΔE*ab, total color difference; B, before washing; A, after washing; I, indigo; JI, Jeju indigo; β-CD, β-cyclodextrin; HP-β-CD, hydroxypropyl-β-cyclodextrin; Control, indigo dyed without cyclodextrin

ΔE*_ab_ $$\:=\sqrt{{\left({L}_{after}^{*}-{L}_{before}^{*}\right)}^{2}+{\left({a}_{after}^{*}-{a}_{before}^{*}\right)}^{2}+{\left({b}_{after}^{*}-{b}_{before}^{*}\right)}^{2}}$$

Dye fixation analysis (Table 3) further supported these findings. The fixation rate of indigo was calculated using Eq. (2). The fixation efficiency of indigo was significantly enhanced by cyclodextrin inclusion, with I:HP-β-CD exhibiting the highest fixation rate (78.06%), followed by I:β-CD (50.81%) and JI:HP-β-CD (45.89%), compared to the control (25.01%). This indicates that cyclodextrin complexes improve dye retention on cotton fibers, likely due to enhanced dispersion and interaction with the fabric matrix.

**Table 3:** Dye fixation (%) of indigo (control) and indigo-cyclodextrin inclusion complexes on cotton fabrics, calculated from color strength (K/S) values measured before and after washing

Samples	K/S before washing (b)	K/S after washing (a)	Dye fixation (%)
Control	0.9002	0.2252	25.01
JI:HPβCD	0.6416	0.2944	45.89
I:β-CD	6.1287	3.1137	50.81
I:HPβCD	5.1107	3.9895	78.06

Overall, these results demonstrate that cyclodextrin inclusion complexes significantly improve dye uptake, color retention, photostability, and fixation efficiency of indigo in aqueous dyeing systems, with I:HP-β-CD consistently showing superior performance due to its enhanced aqueous compatibility, reduced crystallinity, and increased molecular flexibility. This improved performance can be attributed to hydroxypropyl substitution, which enhances cavity accessibility and facilitates more efficient host-guest interactions with indigo. These findings support the bioprocess feasibility of sustainable water-based dyeing using cyclodextrin inclusion complexes derived from renewable starch sources, while avoiding toxic reducing agents such as sodium dithionite that generate hazardous sulfite-rich wastewater. This biocompatible approach leverages the improved aqueous dispersibility and environmental stability of I:β-CD and I:HP-β-CD complexes. Overall, cyclodextrin inclusion enables more environmentally friendly indigo dyeing by minimizing or eliminating hazardous reductants without compromising performance. Further research is recommended to optimize complex formulations and processing conditions, enhance dye penetration and fixation, and explore process scalability or alternative cyclodextrin derivatives for practical eco-friendly textile applications.

## Conclusion

Indigo inclusion complexes with β-CD and HP-β-CD were successfully prepared at a ratio of 1:1 via co-precipitation and kneading. They were characterized using UV–Visible Spectroscopy, FT-IR, PXRD, TEM, and ^1^H NMR, confirming that indigo is encapsulated within the cyclodextrin. Phase solubility analysis revealed an A_L_ type with stability constants (K_s_) of 335 M^−1^ and 450 M^−1^ for the I:β-CD and I:HP-β-CD complexes, respectively. This study showed that indigo aqueous solubility increased 3.58-fold with the I:β-CD complex and 5.55-fold with the I:HP-β-CD complex, both prepared via co-precipitation. In addition to solubility enhancement, the inclusion complexes enhanced thermal stability, particularly the I:HP-β-CD complex, which retained ~ 9 µg/mL indigo after 7 days at 100 °C and showed minimal photodegradation under UV light. This superior performance of HP-β-CD complex can be attributed to the hydroxypropyl substitution, which imparts higher aqueous compatibility, reduced crystallinity, and greater molecular flexibility to the cyclodextrin host. The hydroxypropyl groups disrupt intermolecular hydrogen bonding between cyclodextrin molecules, leading to improved cavity accessibility and more efficient host-guest interactions with indigo. Collectively, these structural characteristics contribute to enhanced complex stability, improved solubilization efficiency, and increased thermal and photostability of the encapsulated dye.

Moreover, the dyed cotton fabrics exhibited distinct color properties and retention behavior, as supported by CIELAB parameters (ΔE*ab), light exposure studies, and dye fixation analysis, demonstrating the effectiveness of cyclodextrin inclusion complexes in improving dye stability and retention. Importantly, the same cyclodextrin-assisted strategy was applicable to natural indigo, enabling its aqueous use without chemical reduction and demonstrating the broader sustainability potential of the system, although the resulting coloration was relatively less intense compared to synthetic indigo complexes. Cyclodextrin-assisted indigo dyeing can reduce the need for harmful reducing agents, and future work could focus on increasing dye uptake, improving uniformity, and using greener additives to enhance dye fixation and advance the commercial viability of eco-friendly dyeing methods.

## Supplementary Information

Below is the link to the electronic supplementary material.

Supramolecular Encapsulation of Indigo using β-Cyclodextrins and Hydroxypropyl-β-cyclodextrin : A Sustainable Strategy for Enhanced Solubility, Stability, and Reducing Agent-Free Dyeing Processes

## Data Availability

Data supporting the findings of this study are included within the article and its supplementary files.
