# Supplementary material for: Supramolecular encapsulation of indigo with β-cyclodextrin and Hydroxypropyl-β-cyclodextrin: a sustainable strategy for enhanced solubility, stability, and reducing agent-free dyeing processes
Source: Bioresour Bioprocess. 2026 May 13;13(1):71. doi: 10.1186/s40643-026-01065-w (PMC13172062; doi:10.1186/s40643-026-01065-w)
Supplement: Supplementary file 1 — Supramolecular Encapsulation of Indigo using β-Cyclodextrins and Hydroxypropyl-β-cyclodextrin : A Sustainable Strategy for Enhanced Solubility, Stability, and Reducing Agent-Free Dyeing Processes [file 40643_2026_1065_MOESM1_ESM.pdf]

Supramolecular Encapsulation of Indigo using β-Cyclodextrins: A Sustainable Strategy for Enhanced Solubility, Stability, and Reducing Agent-Free Dyeing Processes

Koijam Monica Devi[a], Nimya Krishnan[a], Chan-Seo Yeo[a], Kwon-Young Choi[a,b,*]

[a]Department of Molecular Science and Technology, Ajou University, Suwon, Gyeonggi-do, Republic of Korea

[b]College of Advanced Bio-Convergence Engineering, Ajou University, Suwon, Gyeonggi-do, Republic of Korea

Correspondence

Kwon-Young Choi

[a]Department of Molecular Science and Technology, Ajou University, Suwon, Gyeonggi-do, Republic of Korea

[b]College of Advanced Bio-Convergence Engineering, Ajou University, Suwon, Gyeonggi-do, Republic of Korea

Tel.: +82-31-219-1825

E-mail:kychoi@ajou.ac.kr

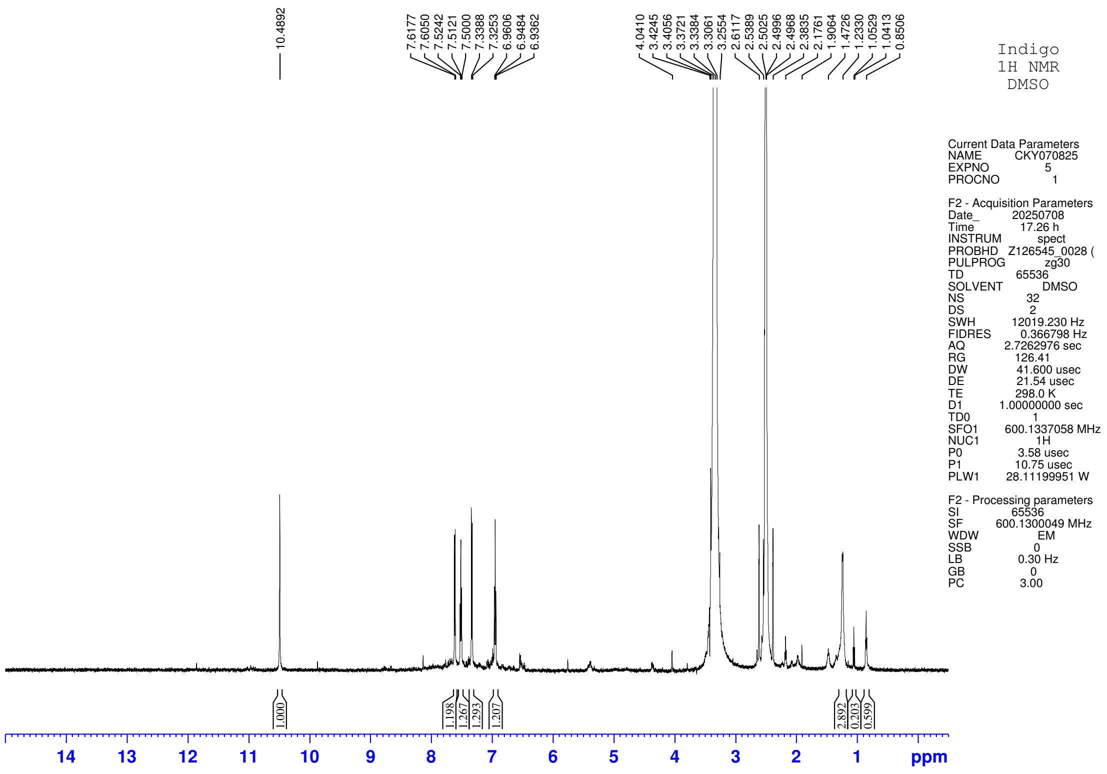

Figure S1: ^{1}HNMR of Indigo.

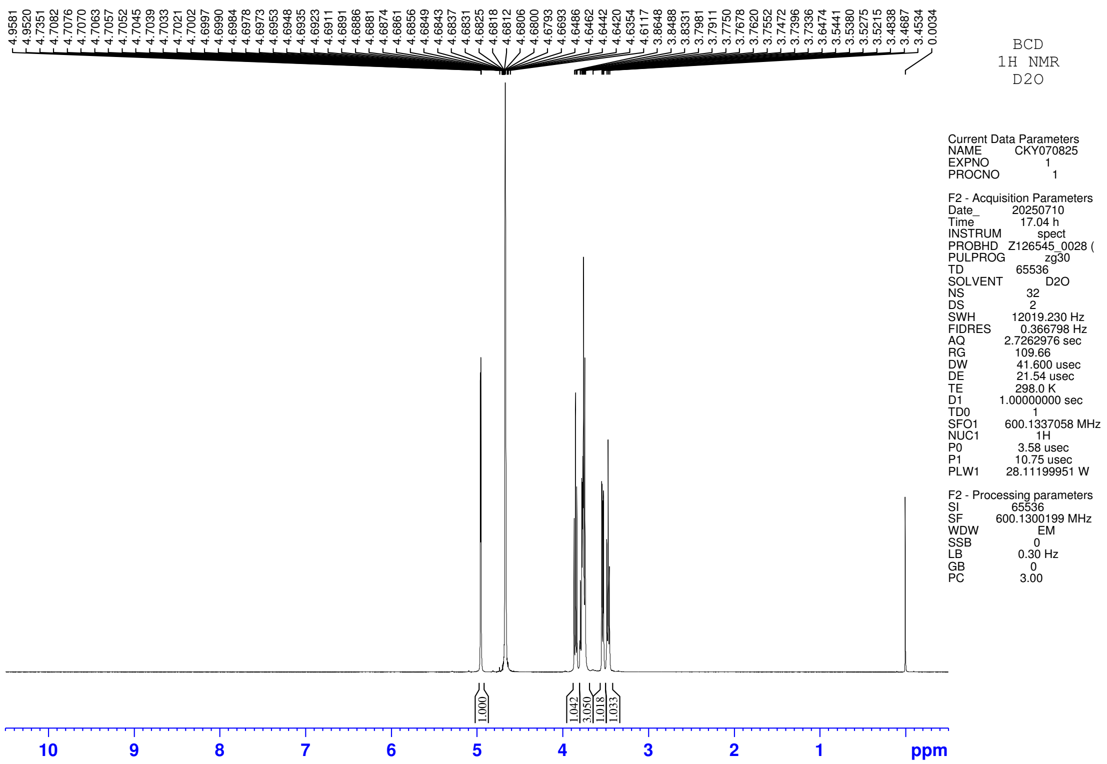

Figure S2: ^{1}HNMR of β-cyclodextrin (β-CD).

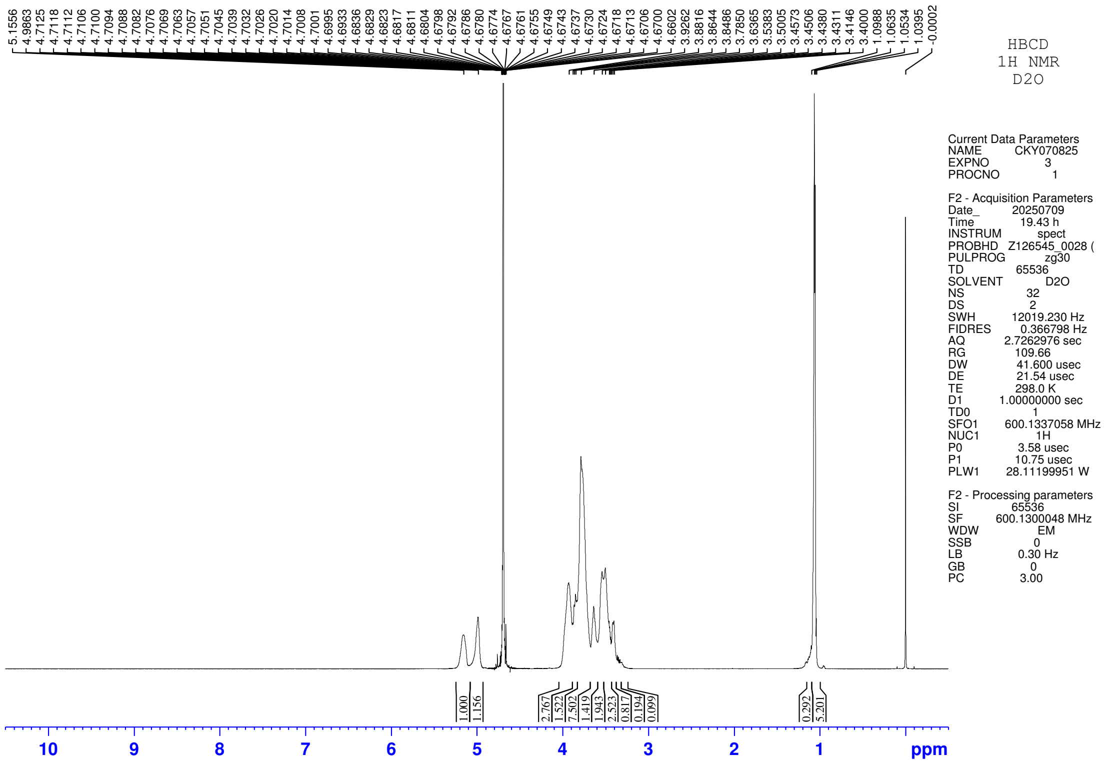

Figure S3: ^{1}HNMR of Hydroxypropyl-β-cyclodextrin (HP-β-CD).

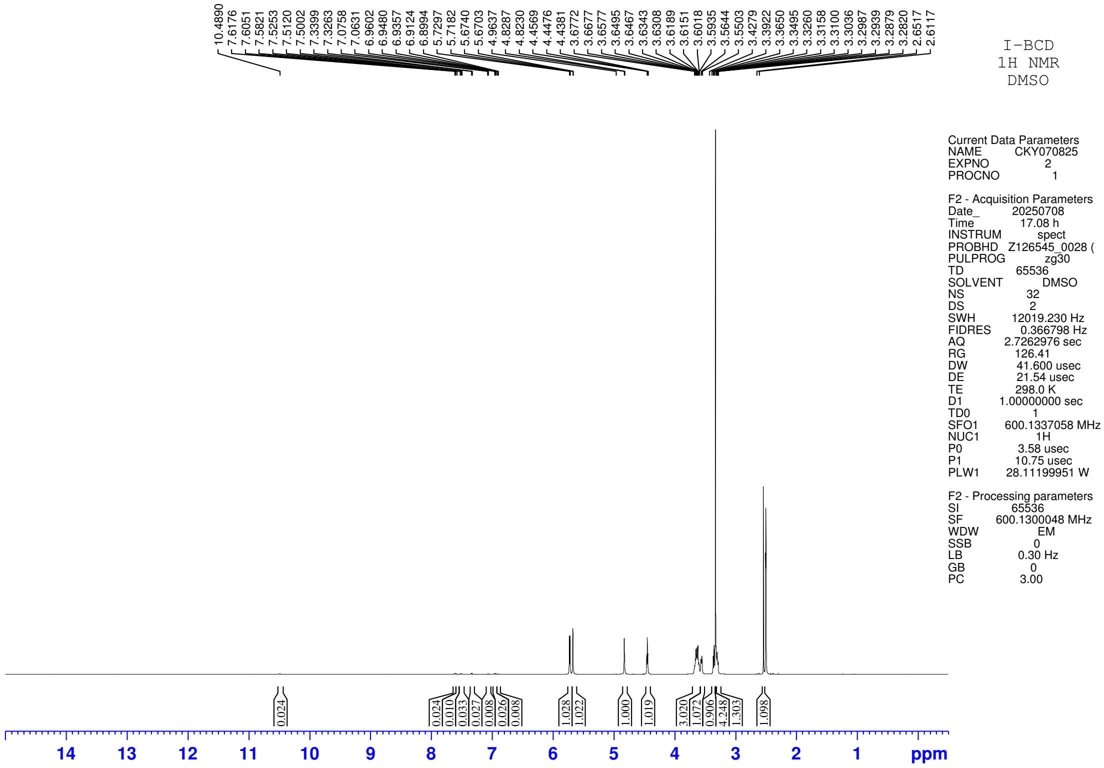

Figure S4: ^{1}HNMR of I: β-CD inclusion complex prepared by co-precipitation.

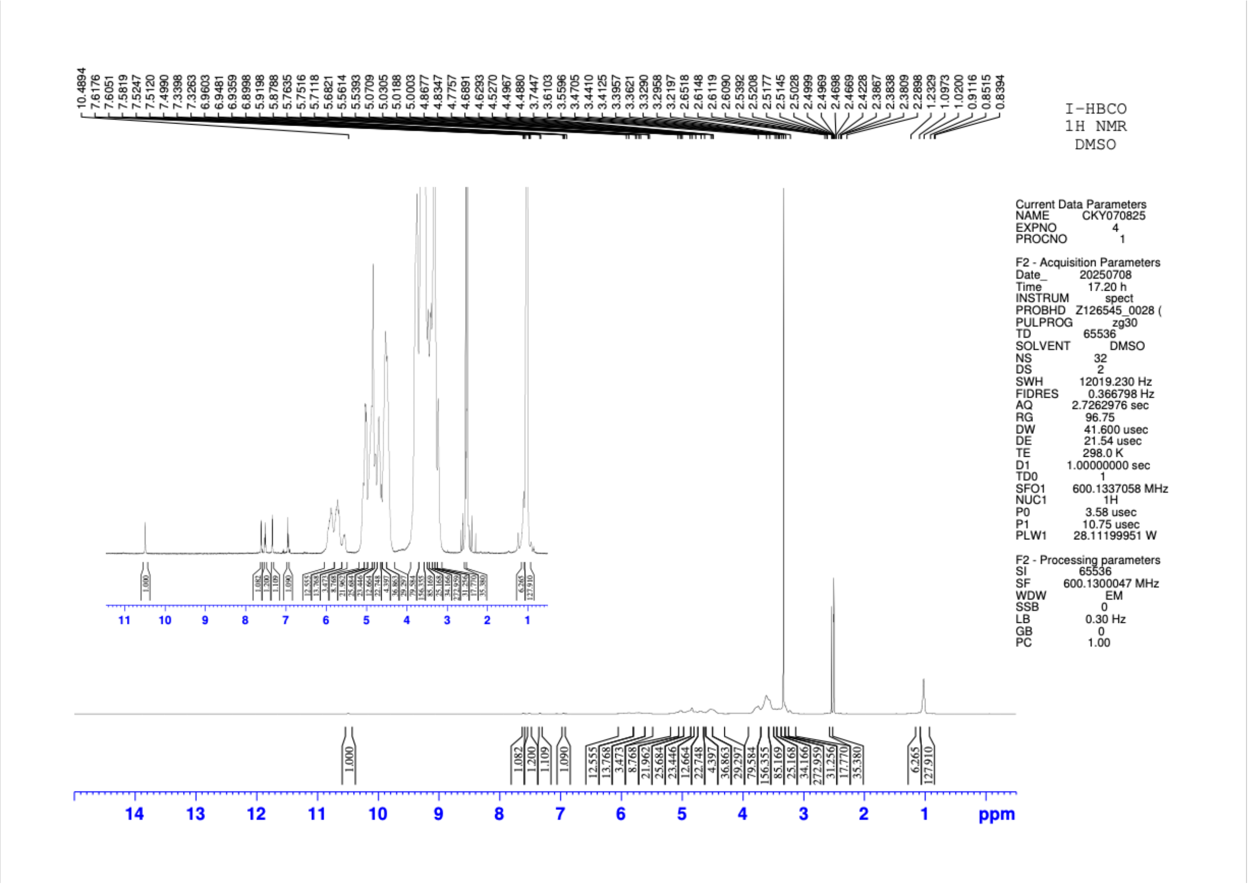

Figure S5: ^{1}HNMR of I: HP-β-CD inclusion complex prepared by co-precipitation.
